# Public health capacity development in Africa: The case of advanced public health education and training in Cabo Verde

**DOI:** 10.7189/jogh.14.03025

**Published:** 2024-08-16

**Authors:** Paulo Ferrinho, António Pedro Delgado, Maria da Luz Lima Mendonca, Inês Fronteira, Mohsin Sidat, Deisa Semedo, Francisca Freyre Monteiro, Ana Cristina Garcia, Pedro Serrano, Manuel Lapão, Dilma Miranda Pires, Lara Ferrero Gómez, Elen Rose Castanheira, Isabel Inês Araújo

**Affiliations:** 1 Global Health and Tropical Medicine, Associate Laboratory in Translation and Innovation Towards Global Health, LA-REAL, Institute of Hygiene and Tropical Medicine, Nova University of Lisbon, Lisbon, Portugal; 2 Faculty of Sciences and Technology, University of Cabo Verde, Praia, Cabo Verde; 3 National Institute of Public Health, Praia, Cabo Verde; 4 National School of Public Health, Public Health Research Centre, Comprehensive Health Research Centre, Nova University of Lisbon, Lisbon, Portugal; 5 Faculty of Medicine, University Eduardo Mondlane, Maputo, Moçambique; 6 Executive Secretariat of the Community of Portuguese Language Countries, Lisbon, Portugal; 7 Center for Research, Institutional Relations and Advanced Training, Capeverdian Jean Piaget University, Praia, Cabo Verde; 8 Faculty of Medicine, Paulista State University Julio de Mesquita, Botucatu, Sao Paulo, Brasil

The public health context is becoming increasingly more complex, gradually associating traditional public health threats connected with infectious diseases with other health problems. This leads to a triple burden of diseases interrelated with growing threats to health security and new conceptual paradigms to approach them [1–3].

More than any other region, Africa feels the weight of these threats without a competent public health workforce to address them [4]. Underlying these workforce shortages is the poor capacity of most African academic institutions [5]. The African Union Commission and Africa Centres for Disease Control and Prevention have called on governments, multilateral organisations, philanthropies, the private sector, and civil society organisations to support the implementation of Africa’s New Public Health Order. This roadmap includes ‘investment in public health workforce and leadership programs to ensure that Africa has the workforce it needs to address health threats’ as one of its five pillars [6].

16 years ago, Ijsselmuiden et al. [7] drew attention to the insufficient number of public health training programs in sub-Saharan Africa and their limited coverage. Fronteira et al. [8] and Fresta et al. [9] identified the limited capacity for medical training in Portuguese-speaking African countries and the lack of investment in post-graduate education despite the rapid expansion of university training in these countries [10,11].

The problem persists. Since 2021, 32% of the 47 countries in the African Region of the World Health Organization (WHO) still do not have postgraduate public health training programs [1]. Investing in advanced public health workforce training is essential to building and strengthening health research capacity in low- and middle-income countries and achieving universal, high-quality, and safe health care coverage [12].

In this paper, we describe the implementation of advanced public health training (APHT) in Cabo Verde, emphasising its unique development aspects and key sustainability pillars.

## METHODS

We conducted a single holistic case study design [13] based on the experience and information of various professionals, including professors, coordinators, heads of institutions, and others directly involved with the different advanced training programs, academic reports, and analysis of relevant policy, academic and programmatic documents related to public health and the health system of Cabo Verde.

## RESULTS

### Context

With a population of 598 682 (in 2023) [14], Cabo Verde is a small island lower middle-income country in the Afrotropical realm of Macaronesia, independent since 1975. Cabo Verde has been very successful in its health policy, achieving significant development and health progress among sub-Saharan African countries [15,16] but remaining particularly susceptible to mosquito-borne pathogens [17,18].

Cabo Verde (with Mauritius, South Africa, Botswana, and the Seychelles) is one of the sub-Saharan African countries in the advanced stages of demographic transitions [19]. Like other small island developing states, its long coastlines make it vulnerable to a range of climate change impacts, including health impacts [20–23].

Despite previous national health workforce strategies, Cabo Verde lacks an updated workforce strategy, a functional national health workforce observatory [2], and public health competencies, including research skills. The local training capacity is limited as well. The Strategic Plan for Human Resources in Health 2015–20, acknowledged an insufficient number of professionals at all levels of health care provision. In relation to public health, the plan recognised that it was a sector of strategic relevance and that ‘strengthening public health should be an important priority for Cabo Verde’ [24]. This included addressing the needs of an ageing population in a context where neither society nor the health services are prepared to address their specific needs [25]. It also included adaptation of health systems and national health research systems, including their human resources, to deal with climate changes and their consequences, which greatly affect island states such as Cabo Verde.

Delgado et al. [26] traced the development of the medical workforce in Cabo Verde since independence. They mentioned that in 2014 the three medical specialities related to public health (public health, epidemiology, and family medicine) registered only 13 doctors (3% of a workforce of 401 doctors). Most public health practice was attributed to general doctors without specialised training. However, some reported attending ad hoc short technical courses related to specific public health competencies, such as health planning [27]. As of February 2023, 15 (2%) of the 735 doctors registered with the medical council are considered public health specialists, nine of which have already retired (personal communication with Maria da Luz Lima Mendonca, February 2023).

### Case presentation

#### Development of undergraduate university training

Higher education in Cabo Verde began after independence in 1975, with a course for training secondary school teachers [27]. Until then, higher education was exclusively provided abroad, with strong support from the communist bloc countries, mainly the Soviet Union and Cuba. In Europe, Portugal was one of the main places in Cape Verdeans’ higher education.

In 1960 (order number 5/997 of August 27 1960), before independence, a mid-level nursing school was established at the Mindelo Central Hospital on the island of São Vicente to address the ‘need to prepare nursing staff and improve the provision of health care’. After independence, the Cabo Verde Ministry of Health assumed responsibility for the nursing schools in Praia and Mindelo. It continued to train general nurses, equivalent to a mid-level professional course, without awarding degrees. In 2009, after the University of Cabo Verde (Uni-CV) creation and intense partnership work between the Ministry of Health and Uni-CV, the two schools were closed, giving the universities responsibility for training undergraduate professional nurses [28].

**Figure 1:**
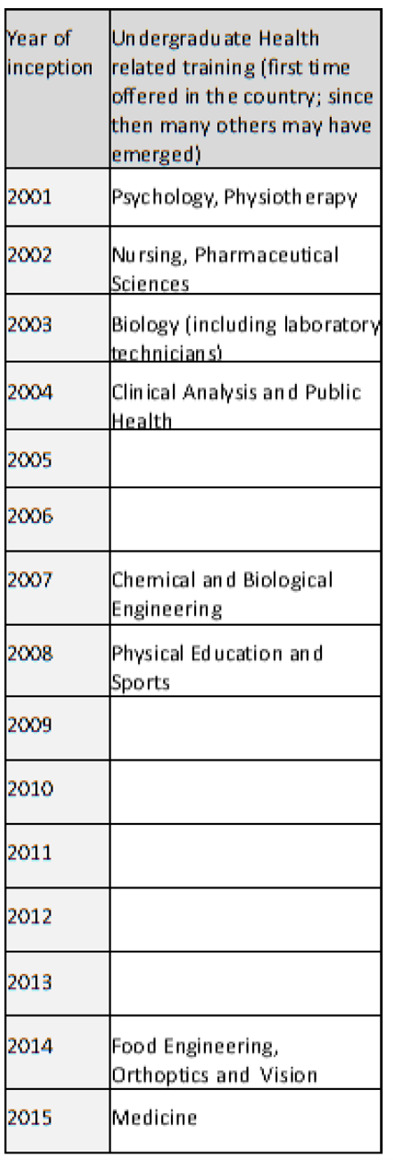
Timeline of the development of health-related undergraduate university education in health sciences in Cabo Verde.

Despite the rapid expansion of the country’s higher education complex [9,10], including for nurses and allied health professionals, doctors were still trained abroad until 2015 [26] (**Figure 1**). Other publications described the skills gap of doctors [29] and the emergence of in-country medical education at the public Uni-CV with the support of the Portuguese University of Coimbra [30–33].

#### Development of post-graduate training

**Figure 2:**
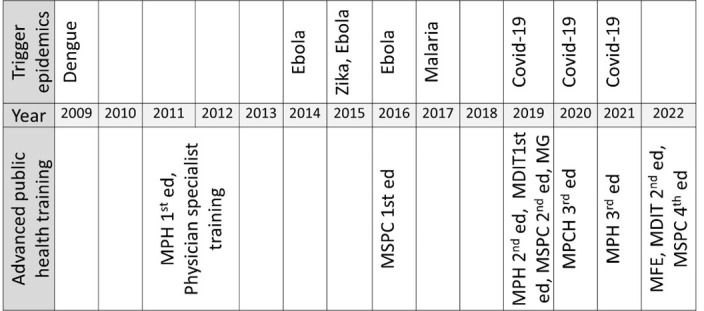
Timeline of the development of advanced public health training in Cabo Verde. ed – edition, MDIT – master in infectious and tropical diseases, MG – master in gerontology, MPH – master in public health, MSPC – master in public and community health.

The first experience of medical specialist training in obstetrics and gynaecology occurred as a once-off program during the 1980s, again with the support of the University of Coimbra (personal communication with Antonio Pedro Delgado, February 2022). **Figure 2** summarises the development of APHT in Cabo Verde.

After a process of planning that started in 2009 during the dengue epidemic that affected the country at the time [34], Uni-CV, with the support of the Paulista State University Julio de Mesquita (Brazil), commenced the first edition of a master’s program in public health in 2011. The program targeted a multidisciplinary audience. This first edition was aligned with the Millennium Development Goals and the National Strategic Plan for Human Resources in Health. It aimed to qualify lecturers for the Uni-CV and expand the country’s public health and research competencies. Most master’s graduates have important roles in the country as academics, researchers, and/or managers of health-related programs, either in the Ministry of Health, other ministries, or even other national and international institutions.

Following a planning process that started in 2007, the first experience of medical specialisation in public health, with the support of the Institute of Hygiene and Tropical Medicine of the Nova University of Lisbon, the National Directorate of Health of Cabo Verde, the Portuguese and Cape Verdean Medical Councils, the Executive Secretariat of the Community of Portuguese Language Countries, the International Institute of Portuguese Language and the Portuguese Language Medical Community, was a once off program during 2011–12. This program had financial support from the African Directorate of WHO and funds from the European Union [9,35]. The course started with eight students from Cabo Verde, three from Guinea-Bissau and one each from Angola, and São Tomé and Príncipe. All doctors trained were recognised as specialists in public health and are playing relevant leadership, service and research functions and roles in their respective countries. This program was donor-driven and not adequately institutionalised within the training academic environment. Hence, after the initial course, no further editions were offered.

Since 2016, in the stride of the 2014–16 Ebola epidemic in West Africa, the 2015 Zika, and 2017 malaria epidemics in Cabo Verde [34,36,37], Uni-CV reformulated the master in public health. This was done with the support of Paulista State University Julio de Mesquita and the Institute of Hygiene and Tropical Medicine of the Nova University of Lisbon. Two blended learning (b-learning) editions were provided, targeting multidisciplinary audiences [38,39]. This reformulation benefitted from Uni-CV’s experience with the Research Centre on Natural Sciences, created in 2021, in 2023 reformulated into the One Health Research Centre. Further, since 2018 the reformulation benefited from the Uni-CV’s experience from the research commitment apparent in the six editions of the Biology and Health Sciences Annual Meetings – JOBS. Once again, all these programs target multidisciplinary audiences.

The private Capeverdian Jean Piaget University followed with a master’s in public and community health with five editions since its inception in 2016, a master’s in infectious and tropical diseases with two editions since its inception in 2019 (the second for students of Guinea-Bissau and Cabo Verde), and a master’s in gerontology, with one edition in 2019 (all in b-learning format since 2019). The master’s in infectious and tropical diseases was created from a solid foundation as a result of the consolidation of the Tropical Diseases Research Group (created in 2012 after the dengue epidemic) to bring evidence in vector-borne diseases to subsidise the public health system in Cabo Verde. Once more, all these programs targeted multidisciplinary audiences.

Following the 2019 joint external evaluation of the International Health Regulations Core Capacities of Cabo Verde [40], the National Institute of Public Health ran four editions of a frontline field epidemiology training program with the support of the West African Health Organization, the Brazilian Association of Field Epidemiologists, Skoll Foundation, the Country Office of WHO, the Centre for Diseases Control of the USA, the Training Programs in Epidemiology and Public Health Interventions Network, and Vital Strategies. Since 2022, with the support of the Africa Centres for Diseases Control, the European and Developing Countries Clinical Trials Partnership, and a network of universities and public health institutes, an advanced training b-learning program in field epidemiology is being offered to a multidisciplinary audience as a master program based at the Uni-CV [39,41–44]. Except for the 2011–12 medical specialisation in public health, all programs were offered in a post-laboral regimen.

## DISCUSSION

This paper addresses the ‘dearth of research about how new training initiatives in public health training’ in Africa come about [45], a necessary step to ensure that academic and other research institutions may be endowed with a competent national academic and health research workforce.

Like other programs in Africa [38,46], APHT in Cabo Verde is academically driven and caters to a broad range of medical, nursing, and allied health professionals working in health services, academia, and other sectors (social, agricultural and environmental sciences, engineering, and law). This contributes to an intellectually rich and challenging learning environment [47].

The efforts to develop APHT capacity in the country have been driven by the public Uni-CV and complemented by efforts from the private sector Capeverdian Jean Piaget University. They follow a partnership approach, which involves strengthening the relationships between national and international organisations [48].

The emergence of information and communication technologies in the country [47–54] and examples from others’ experiences with distance learning [9,46,55–58], together with the constraints resulting from the containment and travel restrictions measures adopted during the coronavirus disease 2019 pandemic, favoured the adoption of b-learning, meeting the recommendations of the Strategic Plan for Education (2017–21) [59]. It also improved access to students residing and working in different islands of this archipelago country and opened the offer to students from other Lusophone countries (Angola, Guinea-Bissau and São Tomé e Príncipe).

APHT in Cabo Verde has developed in response to the demographic transition and public health emergencies in the country and West Africa, as well as the needs identified in strategic plans and external evaluations. Further, APHT developed due to the lack of researchers to staff existing research centres and to develop new ones. Moreover, APHT was developed by the triangulation of efforts from private and public universities, as well as multilateral collaborative networks and public health institutions in the country and abroad. Public health emergencies and subsequent APHT in Cabo Verde contributed to increased laboratory capacity in all inhabited islands of the archipelago, establishing improved testing capability and encouraging integrated responses linking laboratory, clinical, public health units, and research institutions (personal communication with Maria da Luz Lima Mendonça, February 2023).

Epidemics emerged as important drivers for the development of APHT. Most have been associated with zoonoses and vector-borne diseases. Hence, a broader foundation was adopted for APHT, ‘enhancing traditional epidemiology and public health responses with knowledge and skills from a number of areas’ [60]. This led to our inclusion of Capeverdian Jean Piaget University master’s in infectious and tropical diseases in the category of APHT. This program addresses a global deficit in medical entomology capacity, a major focus of the master’s in infectious and tropical diseases [61,62]. As a result of the relentless demographic transition, most of the world’s elderly are in the global south, including Africa. Nevertheless, few health professionals are prepared to address the specific needs of this elderly population [63]. The gerontology master course contributes to reducing this gap.

We acknowledge that ‘training, even if relevant and applicable, makes no more than a latent contribution, one which is activated and realised through alignment of clusters of interacting contextual and relational factors related’ to institutions pertinent to the future career development of the trainees [64]. The sustainability of APHT in Cabo Verde was achievable through institutional capacity development not only of the academic institutions and the universities involved but also through the involvement of employment institutions (the universities themselves and their research centres, as well as the national public health institute and several government departments of animal, plant and environmental health). Further, sustainability was achieved through developing collaborative national consortia (including service providers and professional councils and associations), establishing strong international partnerships and adopting information and communication technologies for distance learning [55]. The reported experiences also reflect the importance of sharing practices between and within low- and middle-income and high-income countries [65]. Above all, they challenge the ‘traditional colonial mindset in global health that expertise flows from North to South,’ allowing students to learn in settings similar to the ones where they will be working in the future and ‘to be exposed to subject experts who have prior experience of working’ in those settings [66].

APHT in Cabo Verde progressed hand-in-hand with research capacity development in a multi-level process that involved, like in other countries, ‘investing in and supporting individuals, teams, organisations (of diverse nature and at several levels), and networks of organisations to increase demand for research, promote researchers’ ability to conduct studies and enable the effective use of findings’ [67]. This is reflected in a sharp increase in the number of health sciences publications from Cabo Verde from 2013 (personal communication with Maria da Luz Lima Mendonça, February 2023).

In the context of Cabo Verde, APHT graduates will have career options that rarely will be purely research-focused and, most likely, will have to combine service provision with research inquiry. This implies that professional career paths of health professionals should value research commitment and consider it one of the criteria for professional promotion and institutional development.

The program that failed to sustain (2011–12 medical specialisation in public health) was donor-driven and not adequately institutionalised within the training academic environment. Hence, after the initial course, further editions were not offered. Successful programs reflect the importance of endogenous capacity development processes [68]. These contributed to research in population health, public policies, and health services evaluation, made scientific studies available to the country, and supported the creation of research groups and the qualification of professionals, lecturers, and researchers.

## CONCLUSIONS

This paper addressed the ‘dearth of research about how new training initiatives in public health training’ in Africa come about. The experience described is particularly relevant to other small island developing states. Like other programs in Africa, APHT in Cabo Verde is academically driven. It caters to various health professionals in health services, academia and other sectors. The fact that students continue working while studying explains the success of post-laboral programs.

Thriving programs reflect the importance of endogenous capacity development processes in dialectic interaction with local and global public health emergencies, progressing hand-in-hand with research capacity expansion in multi-level, multi-sector developments. Information and communication technologies increased the reach of the programs to underserved islands of the country and other small island developing states in Africa.
